# B lymphocyte-typing for prediction of clinical response to rituximab

**DOI:** 10.1186/ar3901

**Published:** 2012-07-06

**Authors:** Hans-Peter Brezinschek, Franz Rainer, Kerstin Brickmann, Winfried B Graninger

**Affiliations:** 1 Department of Internal Medicine, Division of Rheumatology and Immunology, Medical University Graz, Auenbruggerplatz 15, Graz, A-8036, Austria; 2 Internal Medicine, Hospital Barmherzige Brueder, Bergstrasse 27, Graz-Eggenberg, A-8020, Austria

## Abstract

**Introduction:**

The prediction of therapeutic response to rituximab in rheumatoid arthritis is desirable. We evaluated whether analysis of B lymphocyte subsets by flow cytometry would be useful to identify non-responders to rituximab ahead of time.

**Methods:**

Fifty-two patients with active rheumatoid arthritis despite therapy with TNF-inhibitors were included in the national rituximab registry. DAS28 was determined before and 24 weeks after rituximab application. B cell subsets were analyzed by high-sensitive flow cytometry before and 2 weeks after rituximab administration. Complete depletion of B cells was defined as CD19-values below 0.0001 x10^9 ^cells/liter.

**Results:**

At 6 months 19 patients had a good (37%), 23 a moderate (44%) and 10 (19%) had no EULAR-response. The extent of B lymphocyte depletion in peripheral blood did not predict the success of rituximab therapy. Incomplete depletion was found at almost the same frequency in EULAR responders and non-responders. In comparison to healthy controls, non-responders had elevated baseline CD95^+ ^pre-switch B cells, whereas responders had a lower frequency of plasmablasts.

**Conclusions:**

The baseline enumeration of B lymphocyte subsets is still of limited clinical value for the prediction of response to anti-CD20 therapy. However, differences at the level of CD95+ pre switch B cells or plasmablasts were noticed with regard to treatment response. The criterion of complete depletion of peripheral B cells after rituximab administration did not predict the success of this therapy in rheumatoid arthritis.

## Introduction

The use of monoclonal antibodies (mAbs) against cytokines or lymphocyte surface molecules has opened new therapeutic options for patients with rheumatoid arthritis (RA) [1]. By the prediction of a clinical response, these drugs, which are expensive and have the potential for serious toxicity, could be allotted to those patients who would benefit most [2]. B-cell monitoring has been extensively used recently to assess the effect of B cell-directed therapies and the reconstitution of the peripheral blood B-cell repertoire after treatment with the B cell-depleting mAb rituximab. Initially, the clinical response to this therapy was thought not to be correlated to B-cell subset distribution or depletion [3]. This view has been challenged by using high-sensitivity flow cytometry, a technique originally developed to detect small numbers of residual malignant cells. Thus, complete depletion of B cells 2 weeks after the first infusion has been suggested to be an indicator for therapy responsiveness [4-6]. Furthermore, subsequent articles indicated that complete depletion is also a prognostic factor for re-treatment [5] and efficacy of the rituximab therapy [6].

Several articles have analyzed the changes in B-cell subsets following depletion therapy with rituximab [7-9]. In most articles, B cells were characterized by the surface markers IgD, CD27, CD38, and CD24, which allow separation of newly generated 'transitional' (IgD^+^, CD27^-^, CD24^hi^, and CD38^hi^) [10], naïve (IgD^+ ^and CD27^-^), pre-switch (IgD^+ ^and CD27^+^) and post-switch (IgD^- ^and CD27^+^) memory, and double-negative B (IgD^- ^and CD27^-^) cells and plasmablasts (IgD^- ^and CD27^++^) [11-13] in the peripheral blood.

We set out to further delineate B-cell subsets by using high-sensitivity flow cytometry that might help to characterize RA patients who would benefit from rituximab therapy. We expanded our analysis to the co-stimulatory marker CD80, which had been shown to be a potent regulator of IgG secretion by previously activated B cells [14], and CD95, which had been correlated with disease activity in systemic lupus erythematosus (SLE) [13].

## Materials and methods

### Financial disclosure

This work was funded by an unrestricted grant from Roche (Vienna, Austria). The funders had no role in study design, data collection and analysis, decision to publish, or preparation of the manuscript.

### Patients and controls

Fifty-two patients undergoing *de novo *treatment with rituximab for active RA were included in the national 'B Cell surveillance' registry. The participating clinical rheumatologists from local and remote hospitals judged the need for the routine administration of rituximab. Informed consent was obtained from all patients before entering the study, in accordance with the protocol approved by the local ethics committee of the Medical University of Graz. All patients received two 1,000 mg infusions of rituximab preceded by the administration of 100 mg of prednisolone [15]. The characteristics of all patients are shown in Table 1. Disease activity score using 28 joint counts (DAS28) using the erythrocyte sedimentation rate was determined before and 2 and 24 weeks after rituximab application in order to determine the European League Against Rheumatism (EULAR) response. Peripheral blood samples from 17 healthy donors (15 females and two males; mean age of 64 years) were used to determine the normal range for the different B-cell subsets.

**Table 1:** Baseline characteristics of patients included in this study

Parameter	Responders	Non-responders	*P *value
	(*n *= 42)	(*n *= 10)	
Age in years, mean ± SE	62.7 ± 1.9	60.8 ± 3.1	NS^a^
Female gender, percentage	81.0	60.0	NS^b^
Disease duration in years, mean ± SE	11.7 ± 1.3	16.2 ± 3.9	NS^a^
ESR^c^, mean ± SE	39.2 ± 3.8	43.3 ± 11.8	NS^a^
DAS28-ESR, mean ± SE	5.9 ± 0.2	4.9 ± 0.3	0.016^a^
Lymphocytes^d^, mean ± SE	2,138 ± 174	3,083 ± 23	0.0279^a^
RF-positive, percentage	90.8	100.0	NS^b^
ACPA-positive, percentage	76.9	80.0	NS^b^
Double-seropositive, percentage	71.8	80.0	NS^b^
Double-seronegative, percentage	7.7	0.0	NS^b^
Concomitant MTX usage, percentage	40.4	41.7	NS^b^
Previous sDMARD, mean ± SE	2.6 ± 0.1	2.0 ± 0.3	NS^a^
Previous TNF inhibitors, mean ± SE	1.1 ± 0.1	1.4 ± 0.4	NS^a^
No previous biologics, percentage	24.3	11.1	NS^b^
One previous biologic, percentage	43.2	55.6	NS^b^
Two previous biologics, percentage	27.1	11.1	NS^b^
Three previous biologics, percentage	5.4	22.2	NS^b^
Systemic steroids, percentage	40.0	31.0	NS^b^

^a^*P *values were calculated by using Mann-Whitney test; ^b^*P *values were calculated by using the chi-squared test; ^c^mm per 1 hour; ^d^G per liter (normal range is 1.0 to 4.8). ACPA, anti-citrullinated peptide antibody; DAS28, disease activity score using 28 joint counts; ESR, erythrocyte sedimentation rate; MTX, methotrexate; NS, not significant; RF, rheumatoid factor; sDMARD, synthetic disease-modifying anti-rheumatic drug; SE, standard error; TNF, tumor necrosis factor.

### Lymphocyte phenotyping

Peripheral blood samples were drawn before and 15 days after the first rituximab infusion. Peripheral mononuclear cells were prepared as described [4] and stained with the following antibodies: fluorescein isothiocyanate-labeled IgD, phycoerythrin (PE)-conjugated CD24, allophycocyanin (APC)-conjugated CD27, PE-Cy7-labeled CD38, APC-H7-conjugated CD45, horizon Blue-labeled CD19, pyridine-chlorophyll-protein (PerCP)-conjugated CD3 and CD14, and PE-labeled CD80 and CD95 (all mAbs were obtained from BD Biosciences, Schwechat, Austria). Five hundred thousand CD45^+ ^cells were acquired and analyzed by using a seven-channel flow cytometry (BD Canto II cytometer, Software FACSDiva; BD Biosciences). According to their surface staining in the CD27/IgD blot, B cells were classified as naïve (CD19^+^, IgD^+^, and CD27^-^), pre-switch memory (CD19^+^, IgD^+^, and CD27^+^), post-switch memory (CD19^+^, IgD^-^, and CD27^+^), and double-negative (CD19^+^, IgD^-^, and CD27^-^) cells. Plasmablasts were classified as CD19^+^, IgD^-^, and CD27^++ ^[12,16]. In addition, the intensity of the CD38 expression was determined in the post-switch memory and plasmablast populations since this molecule was recently shown not to be constantly expressed within the populations [16,17]. The distribution of co-stimulatory molecules and the FAS receptor on the different B-cell subsets was determined by replacing CD24 with CD80 or CD95. Complete depletion of B cells was defined as CD19 values of below 0.0001 × 10^9 ^cells per liter. The gating strategy and representative dot blots can be found in Figure S1 of Additional file 1.

Since 41% of the blood samples coming from peripheral hospitals had to be transported for more than 12 hours, we compared 15 samples of patients with RA in a pair-wise fashion (2 and 24 hours after blood letting) and found no significant change in absolute counts of B-lymphocyte subsets after this time.

### Statistical analyses

Data were analyzed for normal distribution in order to decide whether to use parametric or non-parametric tests. Values are expressed as the mean ± standard error of the mean or as median (interquartile range) and were calculated by using GraphPad Prism (version 5.0b; GraphPad Software, La Jolla, CA, USA). Baseline clinical variables and B-cell subsets as predictors of response to the first cycle of rituximab were compared with non-responders by using univariate logistic regression. *P *values of less than 0.05 were considered significant.

## Results

### Characterization of rheumatoid arthritis patients

**Figure 1:**
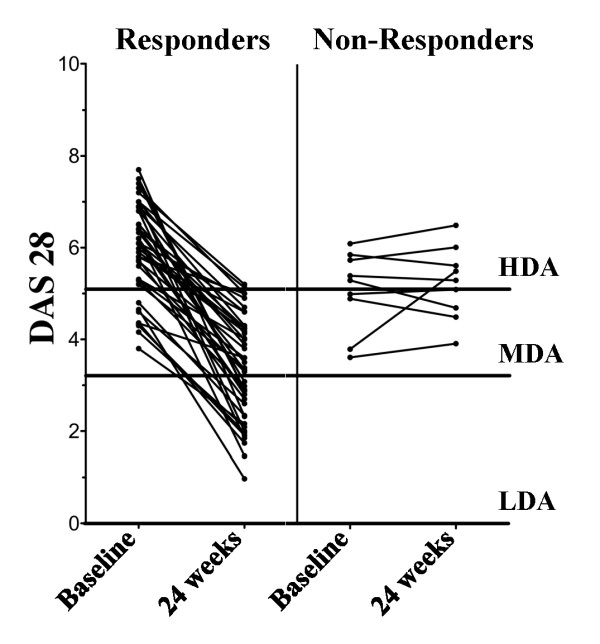
**Disease activity score using 28 joint counts (DAS28) at baseline and 24 weeks after rituximab application in European League Against Rheumatism (EULAR) responders and non-responders**. The lines indicate the border between low disease activity (LDA), moderate disease activity (MDA), and high disease activity (HDA).

Table 1 summarizes the baseline demographics and clinical characteristics of the enrolled patients (*n *= 52), and Figure 1 shows the DAS28 values at baseline and 24 weeks after rituximab application. At baseline, EULAR responders and non-responders were significantly different with regard to the DAS28 only. Interestingly, this difference was due to elevated DAS28 values in RA patients with moderate EULAR response (6.2 ± 0.2; *P *≤ 0.0004), whereas patients with good response were not statistically different from EULAR non-responders (5.7 ± 0.3 versus 4.9 ± 0.3, respectively). In our small cohort, the autoantibody status had no influence on the effect of the treatment with rituximab. The few patients with double-seronegative RA were all in the responder group.

### B-cell depletion and clinical response

**Figure 2:**
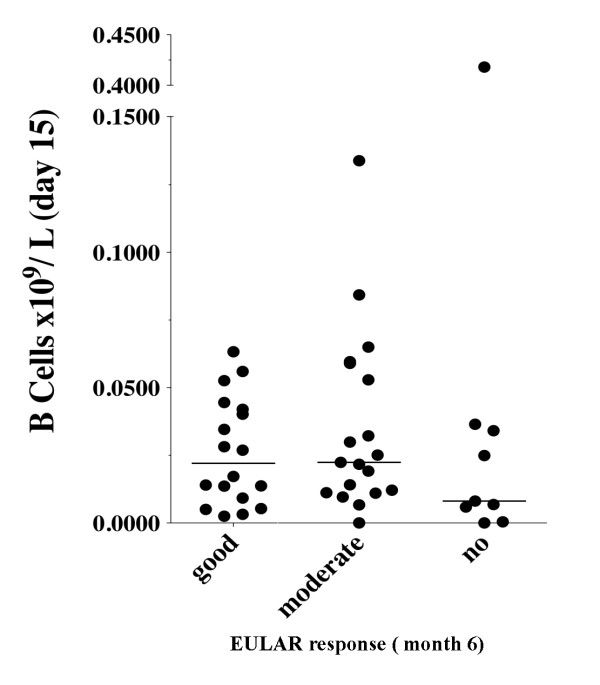
**Number of B cells 15 days after the first rituximab infusion**. Each dot represents one patient. The horizontal lines represent the median.

Two weeks after the first rituximab infusion, there was a dramatic reduction in the number of B cells in all patients with RA. When high-sensitivity flow cytometry was used, the difference in the number of B cells 15 days after rituximab did not reach statistical significance between responders and non-responders. The median numbers (interquartile ranges) of B cells per 10^9^/L were 0.022 (0.008 to 0.043) in RA patients with good EULAR response, 0.022 (0.011 to 0.059) in patients with a moderate response, and 0.008 (0.003 to 0.035) in patients with no response (Figure 2).

### B-cell subpopulations and clinical response

At baseline, the differences in the frequency of naïve, double-negative, pre-switch memory, and post-switch memory B cells between EULAR responders and non-responders did not reach significance. In addition, the expressions of CD80 or CD95 on the B-cell subsets were similar between RA patients and healthy controls. Only a few naïve B cells in patients with RA expressed the co-stimulation and activation markers (1.8% (1.1% to 3.5%) and 4.9% (3.0% to 8.5%), respectively), whereas the highest frequency was found in post-switch memory B cells (58.0% (42.0% to 94.1%) and 65.8% (55.1% to 79.0%), respectively). In all populations analyzed, there was a significant correlation between the frequency of CD95^+ ^and CD80^+ ^B-cell subsets. In responders, this correlation was highest in the double-negative subset (R^2 ^= 0.71; *P *≤ 0.0001) but was lowest in non-responders and controls (R^2 ^= 0.520; *P *≤ 0.0437 and R^2 ^= 0.384; *P *≤ 0.0080, respectively).

**Figure 3:**
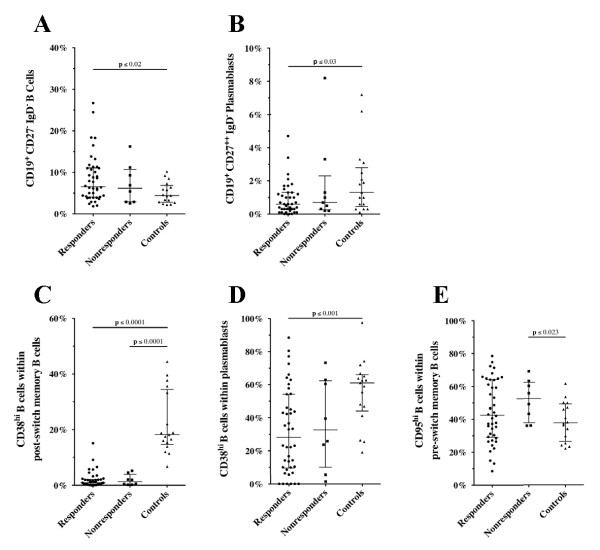
**B cell phenotype distribution before rituximab**. Frequencies of **(a) **CD27^- ^IgD^- ^double-negative B cells and **(b) **CD27^++ ^IgD^- ^plasmablast in patients with rheumatoid arthritis (RA) at baseline and in healthy age-matched controls. The percentages of CD38^hi ^cells within **(c) **the post-switch memory B cells and **(d) **plasmablasts (that is, late plasmablasts [16]) are shown. **(e) **The frequency of CD95^+ ^cells within the pre-switch memory B cells is depicted. Significant differences between the populations using Mann-Whitney-test are indicated. Of note, the difference between non-responders and responders was significant using univariate logistic regression analysis.

When the frequencies of B cells in responders and non-responders at baseline were compared with those in healthy controls, significant dissimilarities were seen (Figure 3). Thus, responders had significantly more double-negative B cells (Figure 3a) and fewer plasmablasts (Figure 3b) in comparison with controls: 6.6% (4.1% to 11.1%) versus 4.4% (2.8% to 6.9%), *P *≤ 0.02 and 0.6% (0.3% to 1.3%) versus 1.3% (0.5% to 2.8%), *P *≤ 0.03, respectively. The frequencies of these B-cell subsets in non-responders were 6.2% (2.1% to 10.7%) and 0.9% (0.3% to 2.8%), respectively.

The total cohort of patients with RA had a significantly lower frequency of CD38^hi ^B cells within the post-switch memory subset (*P *≤ 0.0001). Thus, the median (interquartile range) frequencies of this B-cell subset were 0.9% (0.3% to 2.1%) in responders, 1.3% (0.3% to 3.9%) in non-responders, and 18.2% (14.7% to 34.5%) in controls (Figure 3c). Furthermore, the frequency of CD38^hi ^B cells within plasmablasts was significantly diminished in EULAR responders compared with controls: 28.1% (9.6% to 54.3%) versus 61.1% (44.1% to 66.0%), *P *≤ 0.001 (Figure 3d). In contrast, only non-responders had a significantly higher frequency of CD95^+ ^pre-switch memory B cells than controls: 52.6% (37.9% to 62.6%) versus 37.9% (26.5% to 49.3%), *P *≤ 0.023 (Figure 3e). The frequency of CD95^+ ^cells in the other B-cell subsets is depicted in Figure S2 of Additional file 2. As indicated in Figure 3, no significant differences were found between EULAR responders and non-responders.

### Prediction of first-cycle response

When univariate logistic regression analysis was used (Table 2), only a low frequency of plasmablasts was a valid predictor for EULAR responsiveness (odds ratio of 2.22; *P *≤ 0.04). In our small cohort, complete depletion on day 15 let us calculate an odds ratio of 2.33 for the lack of a therapeutic response to rituximab (*P *= 0.516).

**Table 2:** Baseline clinical variables and B-cell subsets in responders and non-responders, as determined by univariate logistic regression

**Clinical variable or B**-**cell subset**	Responders	**Non**-**responders**	OR (95% CI)	*P *value
Age in years, median (interquartile range)	62.9 (58.2-67.7)	60.6 (49.7-71.4)	0.99 (0.92-1.05)	0.647
sDMARD use, median (interquartile range)	2.5 (2.1-2.8)	2.4 (1.5-3.3)	0.84 (0.33-2.14)	0.956
Prior anti-TNF use, median (interquartile range)	1.1 (0.8-1.4)	1.1 (0.3-2.0)	1.02 (0.37-2.80)	0.978
ESR^a^, median (interquartile range)	6.1 (5.4-6.8)	5.0 (2.9-7.2)	0.74 (0.47-1.17)	0.202
Rheumatoid factor, number (percentage)				
Positive	27 (90.0)	7 (100.0)	1.91 (0.43-8.32)	0.389^b^
Negative	3 (10.0)	0 (0.0)		
B-cell subset, median (interquartile range)				
Naïve cells^c^	8.9 (8.6-9.3)	9.2 (8.6-9.8)	1.51 (0.56-4.11)	0.416
Post-switch memory^c^	7.4 (7.1-7.6)	7.9 (7.4-8.5)	3.91 (0.93-16.45)	0.063
Plasmablasts^d^	3.8 (3.3-4.4)	5.2 (3.8-6.6)	2.22 (1.04-4.74)	0.040
B-cell depletion, number (percentage)				
Complete	2 (6.7)	1 (14.3)	2.33 (0.18-30.10)	0.516
Incomplete	28 (93.3)	6 (85.7)		

^a^Square root values; ^b^calculated from contingency table, Pearson-Mantel-Haenszel *P *value; ^c^natural log (absolute number of cells); ^d^natural log (absolute number of cells + 1). anti-TNF, anti-tumor necrosis factor; CI, confidence interval; ESR, erythrocyte sedimentation rate; OR, odds ratio; sDMARD, synthetic disease-modifying anti-rheumatic drug.

## Discussion

In our small registry cohort of patients with routinely treated RA, complete depletion of B cells 15 days after the first rituximab infusion was not a prognostic factor for clinical response. We did not find technical explanations for this discrepancy with previous reports [4-6]. The time lag between blood letting and analysis was proven not to be a valid explanation since no significant difference was found between samples that were analyzed within 6 or 24 hours (Figure S3 of Additional file 3). We cannot exclude biological differences between our cohort and the patients in previous reports [4-6].

Interestingly, in the regression analysis, the results for baseline distribution of B-cell subsets in responders and non-responders (Table 2) were similar to those reported by Vital and colleagues [5]. Thus, in both studies, fewer plasmablasts were significantly associated with response to rituximab. These B-cell populations were also significantly less frequent in responders than in healthy controls (Figure 3b, d).

Analyzing the frequency of the major B-cell subsets (that is, naïve, pre-switch and post-switch memory, and double-negative B cells and plasmablasts) in our RA patients before rituximab treatment, we and others did not find a significant difference with healthy age-matched controls [11]. Interestingly, separating the RA population in EULAR responders and non-responders revealed a significantly higher percentage of double-negative (IgD^-^/CD27^-^) B cells in the former group (Figure 3a). In a recent study in healthy older people, this population of B cells was shown to be enriched in exhausted cells [18]. In patients with RA, the humoral immune system already seems to be overstimulated, driving more B cells into the double-negative subset in comparison with healthy controls (Figure 3). The significant result for EULAR responders might be related primarily to the higher number of patients in this group. In these patients (in contrast to patients with SLE [13]), disease activity did not correlate with CD95 expression on double-negative B cells (data not shown).

In all patients with RA, CD38^hi ^post-switch memory B cells were significantly reduced in comparison with healthy age-matched controls (Figure 3c). CD38 is a 45-kDa transmembrane glycoprotein expressed on different human cells, including T and B lymphocytes [17]. Activation of naïve B cells leads to an upregulation of this molecule, and transition of activated B lymphocytes into memory cells is characterized by loss of CD38 [19,20] but this molecule reappears when B cells develop into plasmablasts and plasma cells [21]. Plasmablasts can be separated further into CD38^- ^early plasmablasts and CD38^++ ^late plasmablasts [16]. Interestingly, in synovial tissue of patients with RA, CD38^- ^B cells appear to serve as immunoglobulin-producing effector B cells [22]. Whether the CD38^-/low ^post-switch memory B cells in the peripheral blood of patients with RA are similar to the cells described in the synovial tissue cannot be answered yet. Since the latter cells are enriched in the peripheral blood, it is now possible to perform functional assays and determine whether CD38^- ^B cells are similar to mouse B cells that lack RelB and that are defective in proliferative responses but are able to secrete immunoglobulins and undergo class switching [23].

Recently, treatment with tumor necrosis factor (TNF) blockers was shown to alter the distribution of peripheral blood B cells [24]. Thus, infliximab therapy induces an increase in pre-switch memory B cells. Although the majority of our patients have received TNF inhibitors in the past, we did not find this difference. In contrast, our patients exhibited similarly low median percentages of IgD^+^CD27^+ ^memory B cells (8.6% and 6.8% for responders and non-responders, respectively) as described for patients with long-standing RA (10.4%). In addition, the frequencies of these B-cell subsets in the control groups in this study and in the article by Souto-Carneiro and colleagues [24] were almost identical (14.9% and 15.1%, respectively). It is fascinating to speculate whether the missing increase in pre-switch memory B cells is a surrogate marker for TNF failure, but further studies have to confirm these results.

**Figure 4:**
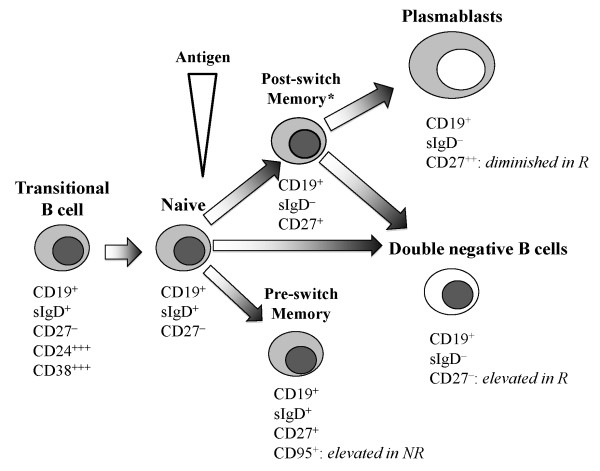
**Model of B-cell differentiation (adapted from **[10,12,16]). *All patients with rheumatoid arthritis (RA) had significantly fewer CD38^+ ^post-switch B cells in comparison with healthy age-matched controls. European League Against Rheumatism (EULAR) non-responders (NR) had a significantly higher frequency of CD95^+ ^pre-switch memory B cells. Rituximab responders (R) had significantly lower levels of plasmablast before treatment and more B cells differentiated into double-negative B cells that are less likely to generate plasma cells [29].

Although pre-switch memory B cells are reduced in our RA cohort, this subset is significantly enriched in CD95^+ ^cells in non-responders (Figure 3e). Similar results were found in patients with SLE [25]. It has been suggested that, in SLE, the pre-switch B-cell population is enriched in autoantibody-producing B cells that are constantly recruited into lymphoid tissue and that therefore are found less often in the peripheral blood. Under physiological conditions, expression of CD95 is important in maintaining peripheral self-tolerance by inducing apoptosis upon engagement with its ligand, CD178 [26]. In RA, defects of this pathway have been demonstrated [27], and accumulation of autoantibody-secreting plasma cells in synovium has been suggested to be the result of the lack of this regulatory mechanism [28]. Our results suggest that an increased frequency of activated (that is, CD95^+ ^pre-switch memory) B cells is associated with poor response to rituximab. Figure 4 summarizes the alterations in the different B-cell subsets between EULAR responders and non-responders compared with healthy age-matched controls. Despite the great efforts made, neither certain cell populations nor serum markers that might help to choose a specific therapy have been found so far. The heterogeneity of the patients with RA (for example, disease duration, previous medication, or co-medication or a combination of these) could skew the results.

## Conclusions

In summary, even in this small cohort, the frequency of plasmablast seems to be the best predictor for response to a B cell-depleting therapy. Still, prospective studies have to confirm this finding. High-sensitivity flow cytometry appears to be very helpful to narrow further candidates.

## Abbreviations

APC: allophycocyanin; DAS28: disease activity score using 28 joint counts; EULAR: European League Against Rheumatism; mAb: monoclonal antibody; PE: phycoerythrin; RA: rheumatoid arthritis; SLE: systemic lupus erythematosus; TNF: tumor necrosis factor.

## Competing interests

The authors declare that they have no competing interests.

## Authors' contributions

HPB and WBG conceived the study and participated in its design and coordination and wrote the manuscript. FR and KB undertook recruitment of patients and collection of clinical data. All authors read and approved the final manuscript.

## Supplementary Material

Additional file 1 **Figure S1 Gating strategy**. Mononuclear cells were incubated in 4 separate tubes containing the following monoclonal antibodies: a) Isotypen-specific control antibodies; b) IgD-FITC, CD27-APC, CD38-PE/Cy7, CD45-APC/H7, CD19-HorizonBlue, CD24-PE, CD3-/CD14-PerCP; c) IgD-FITC, CD27-APC, CD38-PE/Cy7, CD45-APC/H7, CD19-HorizonBlue, CD80-PE, CD3-/CD14-PerCP; d) IgD-FITC, CD27-APC, CD38-PE/Cy7, CD45-APC/H7, CD19-HorizonBlue, CD95-PE, CD3-/CD14-PerCP. CD45 positive leukocytes were gated and after eliminating PerCP positive T cells and monocytes, B cells were separated according to their CD27 and IgD expression. Thereafter the expression of CD38, CD80 or CD95 was analyzed in each B cell population. Click here for file

Additional file 2 **Figure S2 Effect of the time of preparation on the number of day 15 B cells in RA patients with good/moderate or no EULAR response**. Click here for file

Additional file 3 **Figure S3 Frequency of CD95^+ ^cells in the naïve, post-switch and double negative B cell subset**. Click here for file
