# Supplementary material for: B lymphocyte-typing for prediction of clinical response to rituximab
Source: Arthritis Res Ther. 2012 Jul 6;14(4):R161. doi: 10.1186/ar3901 (PMC3580553; doi:10.1186/ar3901)
Supplement: Additional file 1 — Figure S1 Gating strategy. Mononuclear cells were incubated in 4 separate tubes containing the following monoclonal antibodies: a) Isotypen-specific control antibodies; b) IgD-FITC, CD27-APC, CD38-PE/Cy7, CD45-APC/H7, CD19-HorizonBlue, CD24-PE, CD3-/CD14-PerCP; c) IgD-FITC, CD27-APC, CD38-PE/Cy7, CD45-APC/H7, CD19-HorizonBlue, CD80-PE, CD3-/CD14-PerCP; d) IgD-FITC, CD27-APC, CD38-PE/Cy7, CD45-APC/H7, CD19-HorizonBlue, CD95-PE, CD3-/CD14-PerCP. CD45 positive leukocytes were gated and after eliminating PerCP positive T cells and monocytes, B cells were separated according to their CD27 and IgD expression. Thereafter the expression of CD38, CD80 or CD95 was analyzed in each B cell population. [file ar3901-S1.PPT]

## Slide 1
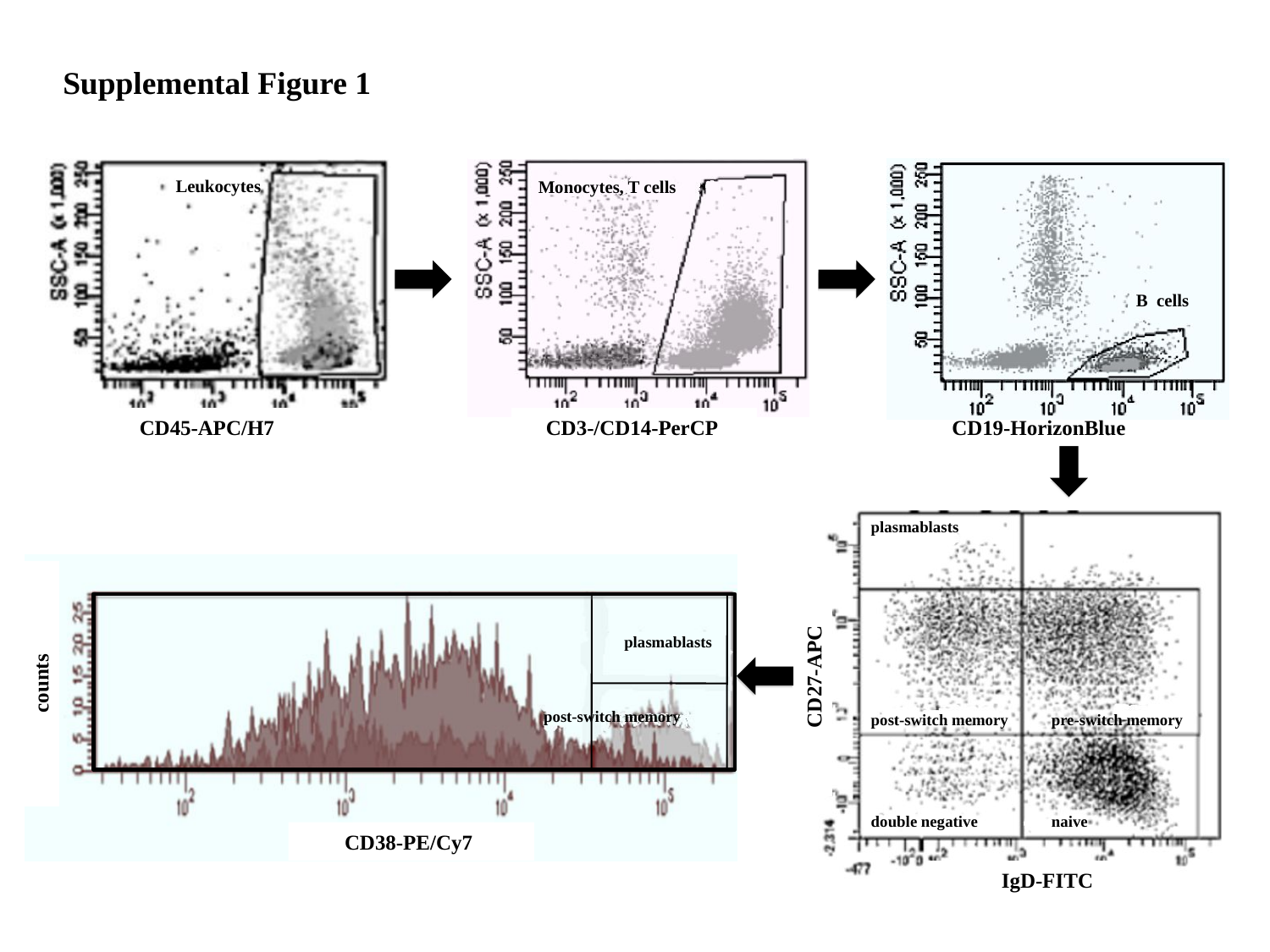

Supplemental Figure 1
Leukocytes
Monocytes, T cells
B cells
CD45-APC/H7
CD3-/CD14-PerCP
CD19-HorizonBlue
plasmablasts
plasmablasts
CD27-APC
counts
post-switch memory
post-switch memory
pre-switch memory
double negative
naive
CD38-PE/Cy7
IgD-FITC
