# Supplementary figures and images for: B lymphocyte-typing for prediction of clinical response to rituximab
Source: Arthritis Res Ther. 2012 Jul 6;14(4):R161. doi: 10.1186/ar3901 (PMC3580553; doi:10.1186/ar3901)

## Slide 1
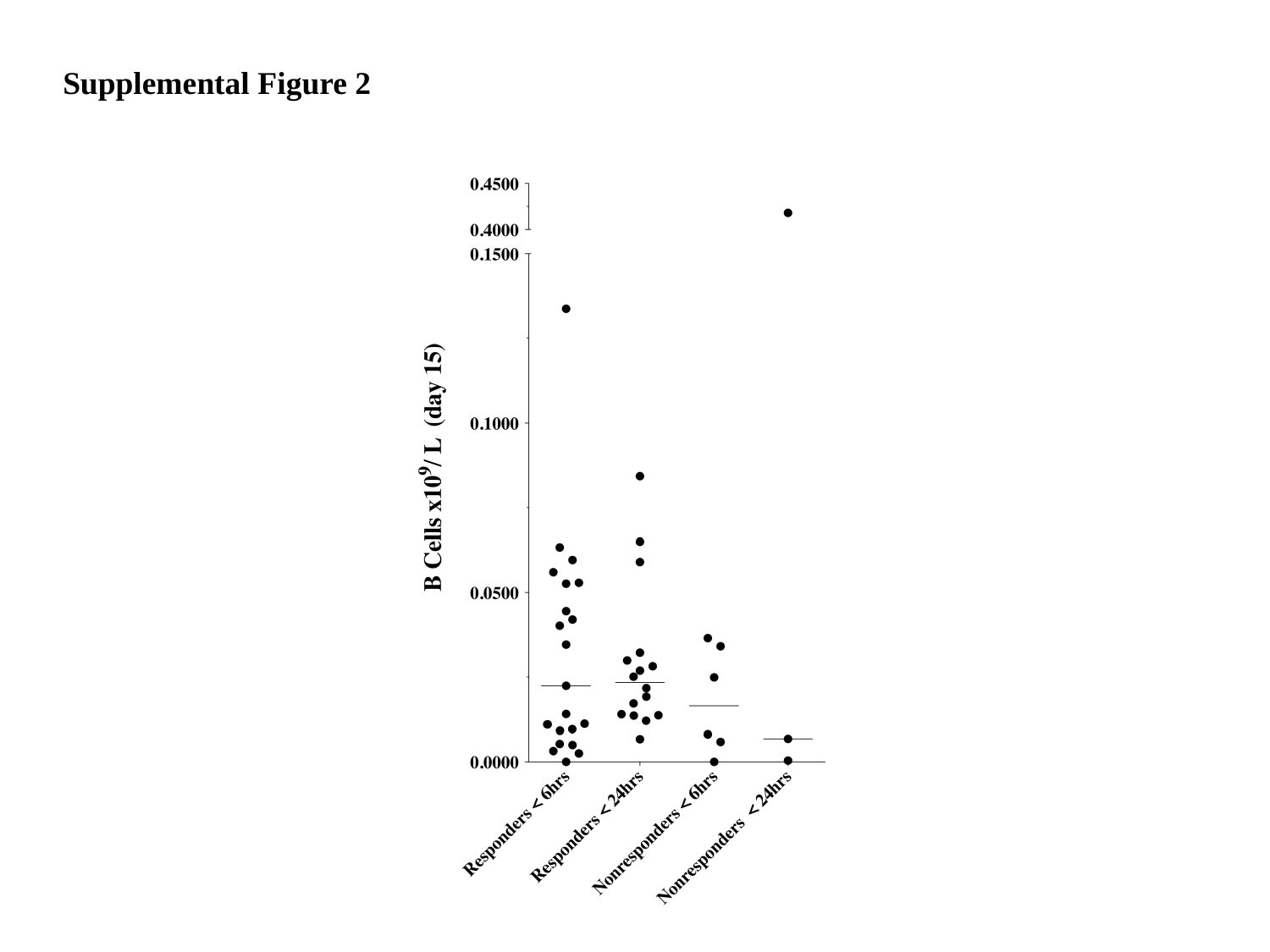

Supplemental Figure 2

Supplement: Additional file 2 — Figure S2 Effect of the time of preparation on the number of day 15 B cells in RA patients with good/moderate or no EULAR response. [file ar3901-S2.PPT]

## Slide 1
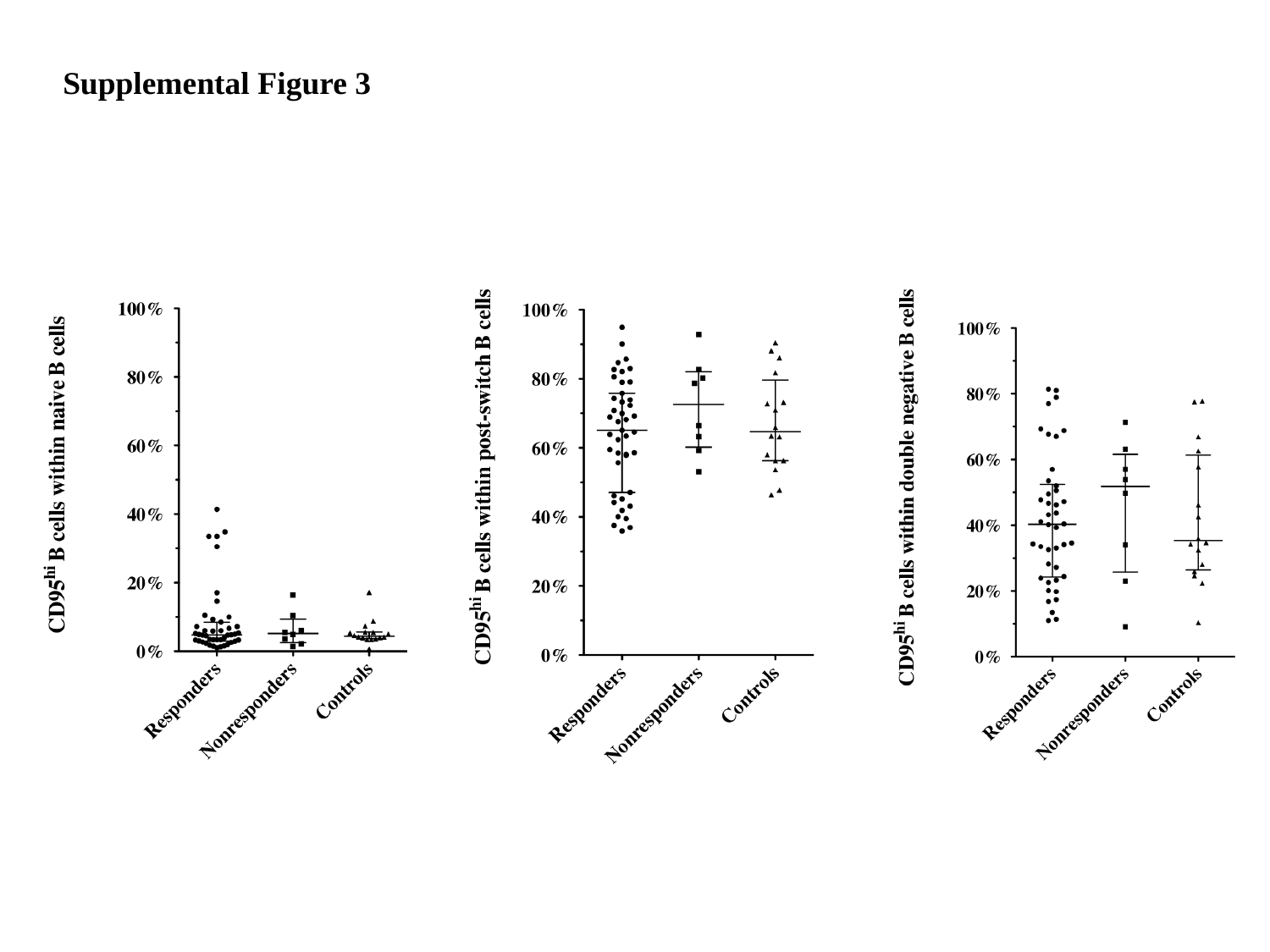

Supplemental Figure 3

Supplement: Additional file 3 — Figure S3 Frequency of CD95+ cells in the naïve, post-switch and double negative B cell subset. [file ar3901-S3.PPT]
